# Glucose and insulin actions after glucose loading on myocardial glucose metabolism in pulmonary hypertension

**DOI:** 10.1093/ehjimp/qyaf044

**Published:** 2025-04-17

**Authors:** Yuki Koga, Nobuhiro Tahara, Yoichi Sugiyama, Shoko Maeda-Ogata, Atsuko Tahara, Munehisa Bekki, Akihiro Honda, Sachiyo Igata, Shuichi Tanoue, Yoshihiro Fukumoto

**Affiliations:** Division of Cardiovascular Medicine, Department of Medicine, Kurume University School of Medicine, 67 Asahi-machi, Kurume 830-0011, Japan; Division of Cardiovascular Medicine, Department of Medicine, Kurume University School of Medicine, 67 Asahi-machi, Kurume 830-0011, Japan; Division of Cardiovascular Medicine, Department of Medicine, Kurume University School of Medicine, 67 Asahi-machi, Kurume 830-0011, Japan; Division of Cardiovascular Medicine, Department of Medicine, Kurume University School of Medicine, 67 Asahi-machi, Kurume 830-0011, Japan; Division of Cardiovascular Medicine, Department of Medicine, Kurume University School of Medicine, 67 Asahi-machi, Kurume 830-0011, Japan; Division of Cardiovascular Medicine, Department of Medicine, Kurume University School of Medicine, 67 Asahi-machi, Kurume 830-0011, Japan; Division of Cardiovascular Medicine, Department of Medicine, Kurume University School of Medicine, 67 Asahi-machi, Kurume 830-0011, Japan; Division of Cardiovascular Medicine, Department of Medicine, Kurume University School of Medicine, 67 Asahi-machi, Kurume 830-0011, Japan; Department of Radiology, Kurume University School of Medicine, 67 Asahi-machi, Kurume 830-0011, Japan; Division of Cardiovascular Medicine, Department of Medicine, Kurume University School of Medicine, 67 Asahi-machi, Kurume 830-0011, Japan

**Keywords:** pulmonary hypertension, myocardial metabolism, glucose, insulin, FDG-PET

A 57-year-old woman with Child-Pugh grade B liver cirrhosis due to autoimmune hepatitis, who has a history of endoscopic variceal ligations for oesophageal varices at the age of 50 and trans-arterial chemoembolization for hepatocellular carcinoma at the age of 56, was referred to our department for preoperative haemodynamic evaluation of a living-donor liver transplantation. The patient was orally treated with branched-chain amino acids, lactulose, ursodeoxycholic acid, and spironolactone. Electrocardiography demonstrated high R wave amplitude in the right-sided precordial leads and deep S wave amplitude in the left-sided precordial leads (see Supplementary data online, *Panel S1*). Chest X-ray showed hilar widening, suggesting pulmonary artery dilatation. Transthoracic echocardiography revealed enlarged right ventricle (RV) with interventricular septal displacement towards the left ventricle (LV) and mild tricuspid regurgitation and estimated systolic pressure of 54 mmHg (*Figure 1A*). Right heart catheterization confirmed the diagnosis of pulmonary arterial hypertension with pulmonary arterial pressure (PAP) of 62/30 (mean 41) mmHg, mean pulmonary arterial wedge pressure (PAWP) of 6 mmHg, cardiac index (CI) of 3.56 L/min/m^2^, and pulmonary vascular resistance (PVR) of 6.14 Wood units. The cause of pulmonary arterial hypertension was identified as portopulmonary hypertension based on a history of oesophageal varices and a hepatic venous pressure gradient of 8 mmHg. After inhalation of highly concentrated oxygen, PAP changed to 57/31 (mean 40) mmHg, PAWP to 11 mmHg, CI to 5.00 L/min/m^2^, and PVR to 3.63 Wood units. PVR decreased by 41% compared with baseline. 18F-Fluorodeoxyglucose (FDG)-positron emission tomography (PET) combined with computed tomography (CT) was performed to evaluate the recurrence and extrahepatic metastasis of hepatocellular carcinoma, and myocardial metabolism. As a result, no recurrence and extrahepatic metastasis were demonstrated and increased glucose metabolism was noted in the biventricular free walls and skeletal muscles (*Figure 1B*). The maximum standardized uptake (SUV_max_) values of the LV and the RV were 4.90 and 8.51, respectively. Plasma glucose level before FDG administration (13 h fasting) was 79 mg/dL, while serum insulin concentration was considerably elevated at 38.7 U/L. We found that she had drank a cup of coffee with sugar 3 h before FDG administration and we decided to redo the examination without glucose loading. Two weeks after the initial PET scan, FDG-PET/CT was assessed on this patient under the same drug regimen to investigate on myocardial glucose metabolism without glucose loading. After a 15 h fasting, glucose and insulin levels were 109 mg/dL and 18.6 U/L, respectively. Intense FDG uptake was not observed in the myocardium and skeletal muscles (*Figure 1C*). The SUV_max_ values of the LV and the RV were 3.20 and 3.38, respectively.

**Figure 1:**
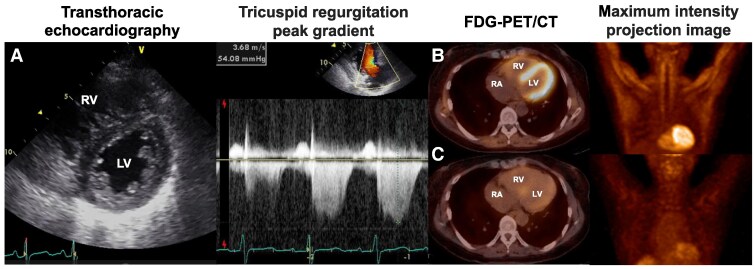
(*A*) Transthoracic echocardiography showing enlarged right ventricle with interventricular septal displacement towards the left ventricle and mild tricuspid regurgitation and estimated systolic pressure of 54 mmHg. (*B*) Increased glucose metabolism was noted in the biventricular free walls and skeletal muscles with glucose loading. (*C*) Increased glucose metabolism was not observed in the myocardium and skeletal muscles without glucose loading.

FDG-PET/CT generates hybrid images of both anatomy and glucose metabolism allowing for an integrated comprehensive body oncology imaging technique. Simultaneously FDG-PET/CT can demonstrate myocardial glucose metabolism. RV glucose metabolism in pulmonary hypertension may be activated by increased RV workload. Glucose and insulin actions have been well-documented in the LV myocardium. However, RV glucose metabolism has not been fully investigated. There is no evidence to support broad conclusions about FDG-PET imaging protocols or myocardial metabolism in pulmonary hypertension. Additionally, there has been no mechanistic investigation into how pulmonary hypertension or liver dysfunction might interact with myocardial glucose metabolism. Liver cirrhosis with portosystemic shunts and/or reduced hepatic mass impairs insulin clearance in the liver, resulting in insulin resistance due to chronic hyperinsulinemia. Insulin resistance reduces tissue response to insulin stimulation, thereby inhibiting glucose uptake in the peripheral muscles and myocardium. This patient had pulmonary hypertension and Child-Pugh grade B cirrhosis with mild hyperinsulinaemia and insulin resistance. Pulmonary hypertension is associated with RV strain, which could affect glucose utilization independently of glucose loading. However, FDG uptakes in the both ventricles were obscure without glucose loading, which might reflect insulin resistance and/or positive pulmonary vasoreactivity.

Caution should be paid against over interpretation of FDG images without confirming patient’s fasting status. In the future, the state-of-the-art study and case series would help to validate these findings. Also, future studies with statistical analyses are warranted to confirm our findings.

## Supplementary Material

qyaf044_Supplementary_Data

## Data Availability

No new data were generated or analysed in support of this research.

## Supplementary data

**Consent:** Written informed consent was obtained from the patient for publication of this case report in line with COPE guidance.

## Lead author biography

Yuki Koga received the MD degree from Kagoshima University (Kagoshima city, Japan) in 2015. Since 2017, he has worked in the Division of Cardiovascular Medicine, Department of Medicine, Kurume University School of Medicine (Kurume city, Japan).

