# Supplementary figures and images for: Glucose and insulin actions after glucose loading on myocardial glucose metabolism in pulmonary hypertension
Source: Eur Heart J Imaging Methods Pract. 2025 Apr 17;3(1):qyaf044. doi: 10.1093/ehjimp/qyaf044 (PMC12034457; doi:10.1093/ehjimp/qyaf044)

## Slide 1
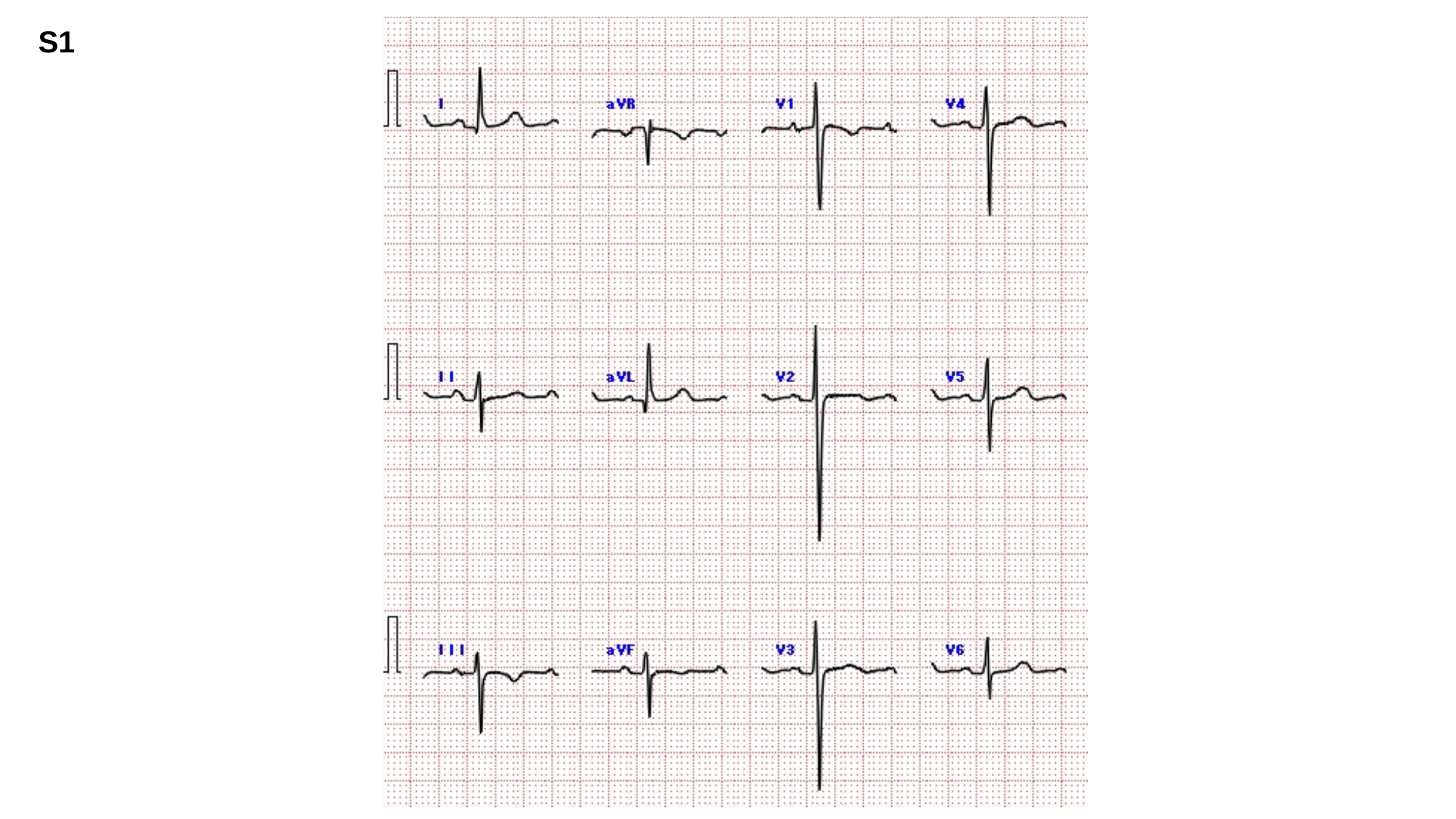

S1

Supplement: qyaf044_Supplementary_Data [file qyaf044_supplementary_data.pptx]
